# Supplementary material for: Caregiver factors influencing family-based treatment for child and adolescent eating disorders: a systematic review and conceptual model
Source: PeerJ. 2025 Apr 9;13:e19247. doi: 10.7717/peerj.19247 (PMC11992973; doi:10.7717/peerj.19247)
Supplement: Supplemental Information 1 [file peerj-13-19247-s001.docx]

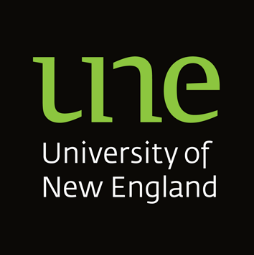
Alex McCord

**School of Psychology**

**University of New England**

Armidale NSW 2351 Australia

smccord@myune.edu.au

**Rationale and Contribution Statement**

Caregiver Factors Influencing Family-Based Treatment for Child and Adolescent Eating Disorders: A Systematic Review and Conceptual Model

The rationale for this review is to capture as broad a range as possible of factors specific to caregivers that influence family-based treatment (FBT) delivery and outcome for child and adolescent eating disorders. Some existing studies have attempted to explore and understand contributing factors to unsuccessful treatment, but have been either more general or narrow than the present review. To date, these studies have focused on evaluating patient and parent factors together (Hagan et al., 2023), combining adult and adolescent outcomes (Gregertsen et al., 2019; Monteleone et al., 2022; Seidinger et al., 2011) or have narrow article inclusion criteria such as a focus on only clinical trials and quantitative studies (Gorrell et al., 2022) or qualitative studies (Cripps et al., 2024). Such studies may review any factors contributing to treatment failure, and while they may capture some caregiver factors, have not been specifically focused on them (Datta et al., 2023) Some studies examine therapy modalities which do not include the intense parental role inherent in FBT, such as enhanced cognitive behavioural therapy (CBTe),adolescent-focused therapy (AFT) or other modalities (Anderson et al., 2021; Hamadi & Holliday, 2020; Vall & Wade, 2015), and others exclude variations of FBT such as multi-family treatment (MFT) or intensive day-treatment programs (DTP) (Gorrell et al., 2022).

A need has been recognised to provide better support to caregivers of young people undergoing FBT, and yet to date, no conceptual model nor framework for assessment exists. This review contains broad inclusion criteria to include evidence ranked by hierarchy from meta-analyses, clinical trials and other quantitative studies, qualitative studies including both caregiver and FBT clinician perspectives, as well as clinical case studies which adhered to the inclusion criteria. We argue that in order to best understand the breadth of caregiver factors which may derail or support treatment, it is vital to include the perspectives of the clinicians to provide the FBT, not only quantitative outcomes.

The unique contribution of this review is the consolidation of results examining one category of variables, caregiver factors, from a wide range of study methodologies, research settings and perspectives. These results of the review were synthesised into a unified framework organised by domain. A further unique contribution is the presentation of the resulting framework in a visual conceptual model using content analysis and frequency of appearance. This model may be tested, used in research settings and may also be used in clinical practice for case formulation and to assist in identifying additional supports tailored to individual families, with the aim of improving recovery rates.

References

Anderson, L. M., Smith, K. E., Nuñez, M. C., & Farrell, N. R. (2021). Family accommodation in eating disorders: A preliminary examination of correlates with familial burden and cognitive-behavioral treatment outcome. *Eating Disorders*, *29*(4), 327-343. https://doi.org/10.1080/10640266.2019.1652473

Cripps, S., Serpell, L., & Pugh, M. (2024). Processes of change in family therapies for anorexia nervosa: a systematic review and meta-synthesis of qualitative data. *Journal of Eating Disorders*, *12*(1), 104. https://doi.org/10.1186/s40337-024-01037-5

Datta, N., Hagan, K., Bohon, C., Stern, M., Kim, B., Matheson, B. E., Gorrell, S., Le Grange, D., & Lock, J. D. (2023). Predictors of family-based treatment for adolescent eating disorders: Do family or diagnostic factors matter? *International Journal of Eating Disorders*, *56*(2), 384-393. https://doi.org/https://doi.org/10.1002/eat.23867

Gorrell, S., Byrne, C. E., Trojanowski, P. J., Fischer, S., & Le Grange, D. (2022). A scoping review of non-specific predictors, moderators, and mediators of family-based treatment for adolescent anorexia and bulimia nervosa: A summary of the current research findings. *Eating and Weight Disorders - Studies on Anorexia, Bulimia and Obesity*, *27*(6), 1971-1990. https://doi.org/10.1007/s40519-022-01367-w

Gregertsen, E. C., Mandy, W., Kanakam, N., Armstrong, S., & Serpell, L. (2019). Pre-treatment patient characteristics as predictors of drop-out and treatment outcome in individual and family therapy for adolescents and adults with anorexia nervosa: A systematic review and meta-analysis. *Psychiatry Research*, *271*, 484-501. https://doi.org/https://doi.org/10.1016/j.psychres.2018.11.068

Hagan, K. E., Matheson, B. E., Datta, N., L'Insalata, A. M., Onipede, Z. A., Gorrell, S., Mondal, S., Bohon, C. M., Le Grange, D., & Lock, J. D. (2023). Understanding outcomes in family-based treatment for adolescent anorexia nervosa: A network approach. *Psychological Medicine*, *53*(2), 396-407. https://doi.org/10.1017/S0033291721001604

Hamadi, L., & Holliday, J. (2020). Moderators and mediators of outcome in treatments for anorexia nervosa and bulimia nervosa in adolescents: A systematic review of randomized controlled trials. *Int J Eat Disord*, *53*(1), 3-19. https://doi.org/10.1002/eat.23159

Monteleone, A. M., Pellegrino, F., Croatto, G., Carfagno, M., Hilbert, A., Treasure, J., Wade, T., Bulik, C. M., Zipfel, S., Hay, P., Schmidt, U., Castellini, G., Favaro, A., Fernandez-Aranda, F., Il Shin, J., Voderholzer, U., Ricca, V., Moretti, D., Busatta, D., . . . Solmi, M. (2022). Treatment of eating disorders: A systematic meta-review of meta-analyses and network meta-analyses. *Neuroscience & Biobehavioral Reviews*, *142*, 104857. https://doi.org/https://doi.org/10.1016/j.neubiorev.2022.104857

Seidinger, F. M., Garcia, C., Böttcher-Luiz, F., & Turato, E. R. (2011). Dropout in the treatment for anorexia nervosa and bulimia: A systematic review from the international databases. *European Psychiatry*, *26*, 738. https://doi.org/https://doi.org/10.1016/S0924-9338(11)72443-9

Vall, E., & Wade, T. D. (2015). Predictors of treatment outcome in individuals with eating disorders: A systematic review and meta-analysis. *International Journal of Eating Disorders*, *48*(7), 946-971. https://doi.org/https://doi.org/10.1002/eat.22411
